# The impact of drainage displacement patterns and Haines jumps on CO_2_ storage efficiency

**DOI:** 10.1038/s41598-018-33502-y

**Published:** 2018-10-22

**Authors:** Ioannis Zacharoudiou, Edo S. Boek, John Crawshaw

**Affiliations:** 1 Qatar Carbonates and Carbon Storage Research Centre, Department of Chemical Engineering, Imperial College London, South Kensington Campus, London, SW7 2AZ United Kingdom; 2 School of Engineering and Materials Science, Queen Mary University of London, Mile End Road, London, E1 4NS United Kingdom

## Abstract

Injection of CO_2_ deep underground into porous rocks, such as saline aquifers, appears to be a promising tool for reducing CO_2_ emissions and the consequent climate change. During this process CO_2_ displaces brine from individual pores and the sequence in which this happens determines the efficiency with which the rock is filled with CO_2_ at the large scale. At the pore scale, displacements are controlled by the balance of capillary, viscous and inertial forces. We simulate this process by a numerical technique, multi-GPU Lattice Boltzmann, using X-ray images of the rock pores. The simulations show the three types of fluid displacement patterns, at the larger scale, that have been previously observed in both experiments and simulations: viscous fingering, capillary fingering and stable displacement. Here we examine the impact of the patterns on storage efficiency and then focus on slow flows, where displacements at the pore scale typically happen by sudden jumps in the position of the interface between brine and CO_2_, Haines jumps. During these jumps, the fluid in surrounding pores can rearrange in a way that prevent later displacements in nearby pores, potentially reducing the efficiency with which the CO_2_ fills the total available volume in the rock.

## Introduction

Multiphase flow is ubiquitous in nature, as well as a plethora of industrial processes. Examples include geological sequestration of CO_2_, enhanced oil recovery (EOR), water infiltration into soil, soil remediation through the removal of liquid pollutants etc. Undoubtedly, there is an inherent complexity in investigating these processes, introduced by the variety of the flow regimes, due to the interplay of the fluids’ affinity to the solid surfaces (wettability)^1,2^ and the complex geometry, where the fluid flow takes place, e.g. permeable media. Compact or non-compact fluid front displacement affects the displacement efficiency, defined as the fraction of the defending fluid that is displaced from the porous rock. For example the non-compact fluid front due to fingering instabilities decreases the displacement efficiency for both EOR and CO_2_ sequestration. Quantifying the displacement patterns is therefore essential for optimizing subsurface processes. A combination of experiments and numerical simulations can elucidate the role of the aspects affecting multiphase flow and help construct upscaled models needed to understand the processes at larger length scales.

Our focus here is on the immiscible displacement of a wetting fluid in permeable media, termed as drainage. Extensive work in the literature addressed the above problem. According to the pioneering work of Lenormand *et al*.^3^ in micromodels, the competition of viscous and capillary forces leads to the basic drainage displacement patterns, namely i) viscous fingering, ii) capillary fingering and iii) stable displacement. These patterns can be mapped on a phase diagram with axes the capillary number *Ca* and the viscosity ratio *M* of the fluids. Since then experimental work in fabricated micromodels^4–8^ and numerical investigations^9–12^ examined the impact of drainage displacement patterns and their domains of validity. Systematic experimental studies covered the impact of wetting conditions^13,14^, pore-scale heterogeneity^15^, as well as the phase of the injected CO_2_^16^. Attention was also given on the crossover between the domains^4–7,17,18^. In micromodels^7^ and in rough fractures^18^ a decrease was observed in the displacement efficiency at the transition zone from viscous fingering to capillary fingering. This trend however hasn’t been observed in three dimensional porous rocks^10^.

Advances in synchrotron-based X-ray computed microtomography enabled the imaging of pore-scale displacement events in porous rocks in real time without disturbing the flow^19,20^. This means that pressure gradients and the viscocapillary balance is maintained during imaging. During slow flow the pore-scale displacement process proceeds not in a smooth continuous way, but as a sequence of sharp interfacial jumps or burst events, called Haines jumps^19–22^. This is reflected by the observed sharp pressure drop in the pressure data (referred as rheons^23,24^). Even though the average flow is at low Reynolds number, inertial effects become important over a transient amount of time during the jump events, with their experimentally observed time scale being around 1–10 ms^25,26^. Haines jumps are associated with both drainage and imbibition dynamics^21,27^, as i) at the draining site the non-wetting fluid passes through a narrow throat displacing the wetting fluid in the wider pore body, while ii) imbibition takes place in surrounding throats with the wetting fluid displacing the injected non-wetting phase. This leads to fluid rearrangement during the jumps, which supplies a significant fraction of the the necessary fluid volume for draining a pore body^19^.

Here we investigate fluid-fluid displacement patterns in a range of fluid flow regimes, by varying the capillary number and the viscosity ratios of the fluids, with the aim of better understanding the implications of these flow regimes on CO_2_ geological sequestration. Supercritical CO_2_ (non-wetting) is being injected deep in geological formations to displace the resident fluid (e.g. brine or oil - wetting fluid) in the pore matrix and to be safely stored there over long times. Understanding the displacement processes taking place at the pore-scale is essential in maximizing the displacement efficiency and it is of paramount importance as CO_2_ geological sequestration appears to be an important tool for combating global warming.

We give particular attention to the low *Ca* flow regime, characterised by Haines jumps, and the impact of the associated fluid redistribution on the displacement process. This aspect has not been thoroughly investigated so far. Experimentally it is not easy to identify the imbibition sites and quantify the degree of fluid redistribution^19,20^; rather this can be assessed from the comparison of the pore filling rates and the feed flow rate of the pump^19^. Numerically, a full Navier Stokes solver is needed to include inertial effects during the jumps. Tsuji *et al*.^10^ investigated drainage in Berea sandstone using the color-gradient lattice Boltzmann method^28^ and applying a body force (pressure gradient) to drive the fluid flow. However, this can be a limiting factor in assessing the displacement efficiency in the low *Ca* flow regime, as decreasing the body force to decrease *Ca* may lead to a pressure difference not sufficient to overcome the capillary entry pressure. In this case no flow is observed and the non-wetting phase is only capable to reach up to a given injection depth in the porous rock^9,10,29^. To the best of our knowledge only Yamabe *et al*.^12^ report numerical work on the impact of Haines jumps on displacement efficiency. They investigated drainage in a synthetic granular rock model, in relation to CO_2_ geological sequestration, and made a distinction between backward and forward Haines jumps. The authors showed that forward flowing events cause a significant drop in CO_2_ saturation, whereas backward flowing events cause an increase of the CO_2_ saturation per injection depth. However, the impact of these pore scale events on CO_2_ displacement efficiency is not clear and believe that this investigation should be extended in several ways:

1. The domain size used by Yamabe *et al*.^12^ is 100^3^ voxels, corresponding to a physical sample size of 1 *mm*^3^. It is not clear whether this domain size and sample resolution used are sufficient. To investigate this point, we extend the domain size to 700^3^ voxels using Graphics Processing Units (GPUs) to accelerate the computations and handle the large numerical load. We also increase the sample resolution, so that the domain size corresponds to a physical sample size of 32 *mm*^3^.
2. Yamabe *et al*.^12^ drive the fluid flow using a body force. Although the authors were not explicit as to how they treat the inlet/outlet boundaries, we suspect that they apply Periodic Boundary Conditions in the direction of the flow. Here we extend this investigation by using a constant injection flow rate to drive the fluid flow (velocity boundary conditions).
3. Given the small domain used by Yamabe *et al*.^12^, in combination with the use of body forcing and Periodic Boundary Conditions, we believe that their results could be affected by boundary effects. Haines jumps at one end may affect the fluid flow at the inlet. Here we investigate this point by using a larger domain size in combination with constant velocity boundary conditions.
4. Yamabe *et al*.^12^ do not report investigations of possible fluid redistribution during Haines jumps. This, however, is a very important aspect of the Haines jump phenomenon^19^. In this paper, we present computational results of extensive fluid redistribution associated with Haines jumps.

Here we demonstrate that numerically we capture the main features of the Haines jumps: i) sharp increase in the non-wetting phase velocity, ii) sharp pressure drop and iii) extensive fluid redistribution. Simulations reveal that Haines jumps can potentially decrease the displacement efficiency of the injected phase, irrespective of the type of the jump, labeled as backward or forward jump event. Irreversible fluid redistribution during the events is essentially responsible for the decrease in the displacement efficiency, as wetting phase, flowing in the direction opposite to the mean direction of the flow, can block the access to other regions of the pore space due to subsequent events affecting the displacement pathways.

## Results and Discussion

**Figure 1:**
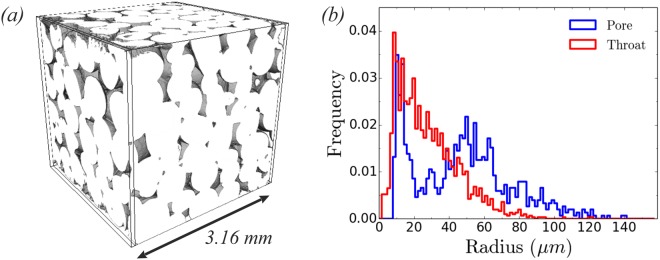
(**a**) Geometry used for the LB simulations reconstructed from micro-CT images of Ketton limestone^30^. The simulation system size is 700^3^ lattice units (l.u) at a resolution of 4.52 *μm* per l.u, which corresponds to a physical system size of 31.6 *mm*^3^, porosity 0.159 and permeability 9.4 Darcy^30^. The Representative Element of Volume, for singe phase flow, using the convex hull approach^58^ is 150^3^ l.u (680 *μm*)^3^. A small reservoir (16 l.u) is added at the inlet/outlet of the simulation domain. (**b**) The pore (blue) and throat (red) size distributions of the Ketton rock sample used in the simulations^59^.

We directly solve the hydrodynamic equations of motion on a three dimensional geometry reconstructed from micro-CT images of Ketton limestone^30^, see Fig. 1, using multi-GPU free energy lattice Boltzmann (LB) simulations^31–33^. The simulation system size is 700^3^ lattice units (l.u) at a resolution of 4.52 *μm* per l.u. (physical system size 31.6 *mm*^3^). Simulations cover a range of viscosity ratios *M* = *η*_nw_/*η*_w_ and capillary numbers *Ca* = *η*_nw_*u*_nw_/*γ*, where *γ*, *η*_*i*_, *u*_*i*_ (*i* = w, nw) are the surface tension, viscosity of the fluids and average fluid velocity respectively. The subscripts w, nw denote the wetting and non-wetting phases. We apply a constant injection flow rate at the inlet/outlet boundaries, i.e. the fluid flow is driven by applying velocity boundary conditions by adopting the approach proposed in^34^ to two-phase flow. The numerical scheme proved to be stable over long simulation times and has been already validated in our work on Haines jumps in simplified micromodels^27^. For each viscosity ratio case we fix *η*_*i*_ (*i* = w, nw) and *γ*, while in order to decrease *M* we increase the wetting phase viscosity *η*_w_. The capillary number varies in the range 8.5 × 10^−6^ ≤ *Ca* ≤ 10^−2^ by varying the volumetric injection flow rate, ${Q}_{inj}={\int }_{A}\,{{\bf{u}}}_{{\rm{nw}}}^{inj}\cdot d{\bf{A}}$, where **A** is the cross sectional area at the inlet/outlet and ${{\bf{u}}}_{{\rm{nw}}}^{inj}$ is the injected velocity of the non-wetting phase ($2\times {10}^{-6}\le {{\bf{u}}}_{{\rm{nw}}}^{inj}\le {10}^{-3}$). Typical values for the ratio of viscous to capillary forces at the pore scale are in the range of 10^−10^–10^−3^, depending on the distance from the injection point in the well bore^35^. We note here that we keep the Ohnesorge number fixed ($Oh={\eta }_{{\rm{nw}}}/\sqrt{\rho \gamma {L}_{s}}\sim 1.3\times {10}^{-2}$) in all simulations, except for two simulations at the highest *Ca* for *logM* = −1 and −2, where for numerical stability reasons we had a slightly higher *Oh*. The Ohnesorge number, which describes the relative importance of viscous forces to inertial and surface tension forces, is fixed for a given experimental system and can be also expressed as *Oh*^2^ = *Ca*/*Re*. For a brine - CO_2_ system, *Oh* is in the range of 10^−3^ to 10^−2^, depending on the characteristic length scale of the system *L*_*s*_, as well as the fluids’ properties^27,36^. *L*_*s*_ is taken to be the average pore throat radius, as this controls the pressure at which pores drain. Finally, the equilibrium contact angle is set to *θ*^*eq*^ = 40°, consistent with contact angle measurements in Ketton at reservoir conditions for a supercritical CO_2_ - brine system^37^.

### Drainage displacement patterns

**Figure 2:**
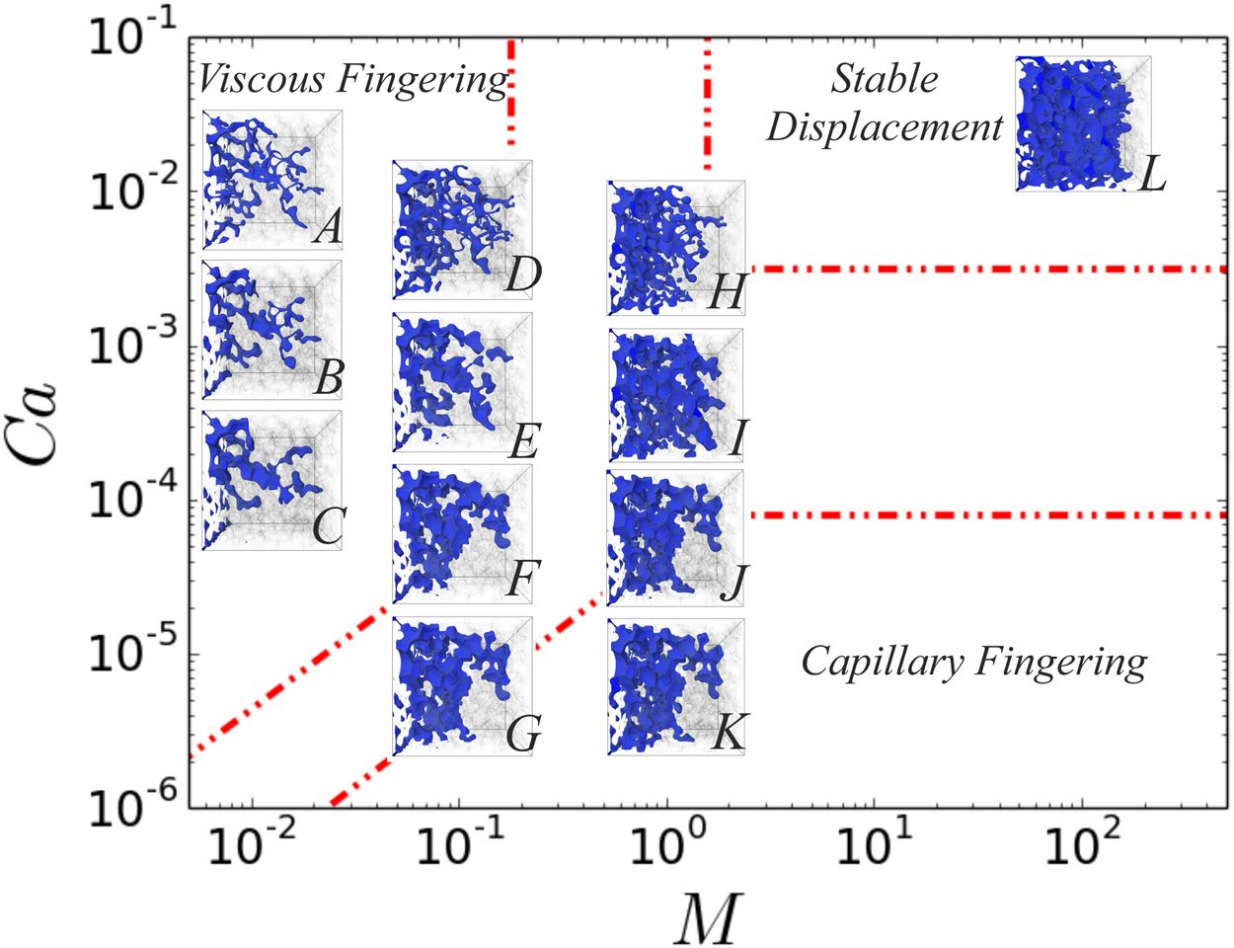
Drainage displacement patterns in the (*Ca*, *M*) phase diagram. The non-wetting phase configuration is shown in blue when it reaches the outlet reservoir (breakthrough), while rock grains and wetting phase are transparent. The three typical fluid displacement patterns are observed, including viscous fingering (high *Ca*, log *M* < 0), capillary fingering (low *Ca*) and stable displacement (high *Ca*, $\mathrm{log}\,M > 0$)^3^. The domain boundaries shown with the red dashed dotted lines are from the experimental work of Zhang *et al*.^6^.

A qualitative demonstration of the displacement patterns in the (*Ca*, *M*) phase diagram is shown in Fig. 2, where only the non-wetting phase is shown when it reached the outlet reservoir. We verify the existence of the three typical fluid displacement patterns, namely viscous fingering (high *Ca*, ${\rm{l}}{\rm{o}}{\rm{g}}\,M < 0$), capillary fingering (low *Ca*) and stable displacement (high *Ca*, $\mathrm{log}\,M > 0$)^3^, with the main focus given on the first two. Our aim is to examine the impact of the displacement patterns on the non-wetting phase saturation: i) when the non-wetting phase reaches the outlet (breakthrough), ${S}_{{\rm{n}}{\rm{w}}}^{br}$, as well as ii) the maximum non-wetting phase saturation, ${S}_{{\rm{nw}}}^{max}$, obtained if the injection continues beyond the breakthrough point. The latter determines the maximum achievable displacement efficiency of the injected phase, which in real porous media can reflect encountering a fracture or a higher permeability zone.

**Figure 3:**
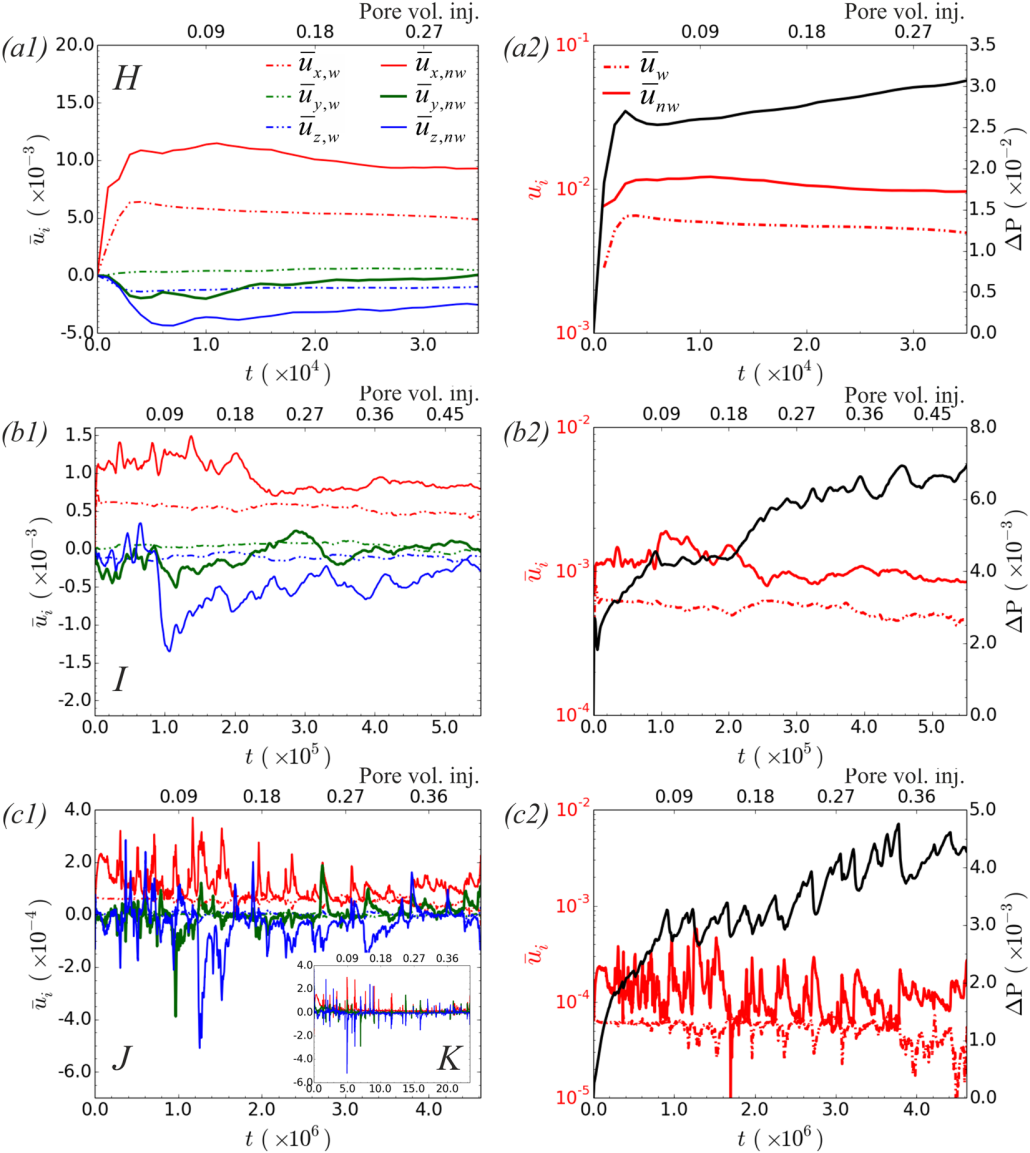
Results from simulations reported in Fig. 2 ($\mathrm{log}\,M=0$): (**a**) *Ca* = 3.0 × 10^−3^ (*H*), (**b**) *Ca* = 3.1 × 10^−4^ (*I*) and (**c**) *Ca* = 3.8 × 10^−5^ (*J*) up to breakthrough time (*t* ≤ *t*_*br*_). Left panel: Average value for the components of the velocity for the wetting/non-wetting phases (dashed/solid lines). Inset of (c1): *Ca* = 1.0 × 10^−5^ (*K*). Right panel: The corresponding average magnitude of the velocity and inlet/outlet pressure difference.

Fig. 3(a1–c1) shows the average value for the three components of the velocity for the wetting/non-wetting phase for simulations with $\mathrm{log}\,M=0$ and varying *Ca*; the right panel, Fig. 3(a2–c2), shows the corresponding average magnitude of the velocity and the inlet/outlet pressure difference, Δ*P* = *P*_*inlet*_ − *P*_*outlet*_. The mean direction of the flow is in the *x*-direction. As *Ca* decreases a distinct change in the flow field marks the transition to the capillary fingering regime. Sharp interfacial jumps, indicative of Haines jumps, become profound and lead to a significant increase of the fluids velocity, see Fig. 3(c). The occurrence of Haines jumps is also evident from the abrupt pressure drop observed in Fig. 3(c2), which coincides with the interfacial jumps.

### Non-wetting phase saturation Vs Drainage displacement patterns

**Figure 4:**
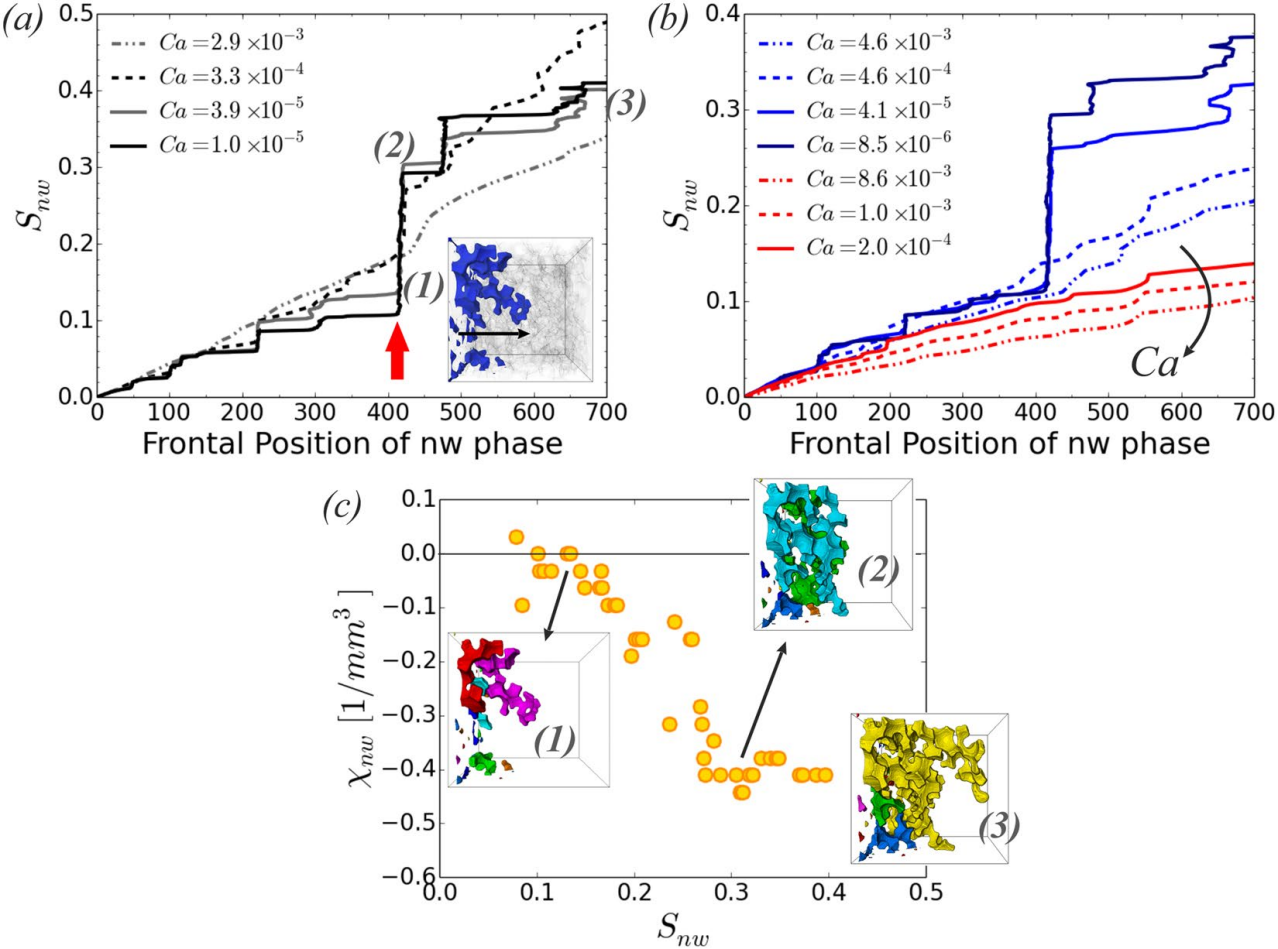
The non-wetting phase saturation, *S*_nw_, as a function of the frontal position, defined as the maximum distance from the inlet - see inset of (**a**), for simulations with varying *Ca* and viscosity ratios: (**a**) $\mathrm{log}\,M=0$ (Fig. 2H–K), (**b**) $\mathrm{log}\,M=-\,1$ (Fig. 2D–G, blue lines) and $\mathrm{log}\,M=-\,2$ (Fig. 2A–C, red lines). The red arrow indicates a position of high capillary entry pressure for moving forward (*x*-direction). (**c**) The Euler characteristic *χ*_nw_, normalized with the total volume, for *Ca* = 3.9 × 10^−5^, $\mathrm{log}\,M=0$ (Fig. 2J). The non-wetting phase configuration for the labelled points (1)–(3) in (**a**), with different colors denoting the disconnected clusters, visually demonstrates the increase in non-wetting phase connectivity.

Examining the non-wetting phase saturation, *S*_nw_, as a function of the frontal position, see Fig. 4(a,b), provides useful information about the type of dispacement. The frontal position is defined as the distance from the inlet of the most advanced tip of the non-wetting phase. High *Ca* results in a linear increase of *S*_nw_ with the frontal position of the injected non-wetting phase. As *Ca* decreases (transition towards capillary fingering) a distinctive step-like structure emerges. Similar behavior is observed for $\mathrm{log}\,M < 0$ (Fig. 4(b)) which favors viscous fingering at high *Ca*, with the transition from viscous to capillary fingering occuring at a lower *Ca*. The step-like structure observed for capillary fingering displacement is due to the sequential forward and backward Haines jump events. A forward Haines jump (or sequence of events) will increase the frontal position significantly, while *S*_nw_ under the time scale of the jumps will not increase significantly. Rather what is happening is that the neighbouring regions will provide the non-wetting phase needed for filling a wider pore space through fluid rearrangement. When the interface reaches a point where the capillary entry pressure for moving forward is higher than the other available displacement pathways, for example the location indicated with the red arrow in Fig. 4(a), then jump events will be observed in the *y* and *z* directions, perpendicular to the mean flow direction, as well as in locations further behind the frontal position. This leads to significant increase of *S*_nw_ (Fig. 4(a) 1 $\to$ 2). The capillary fingering displacement pattern leads to loops of the non-wetting phase towards the inlet and enhances the connectivity of the non-wetting phase, as evident in Fig. 4(c) from the reduction in the Euler characteristic *χ*_nw_^38,39^. Visually this is also shown in the subfigures with different colours denoting the disconnected clusters of the non-wetting phase. We measure *χ*_nw_ using a robust tool in Matlab^40^ and normalize it with the total volume to describe it as a density in physical units [*mm*^−3^].

**Figure 5:**
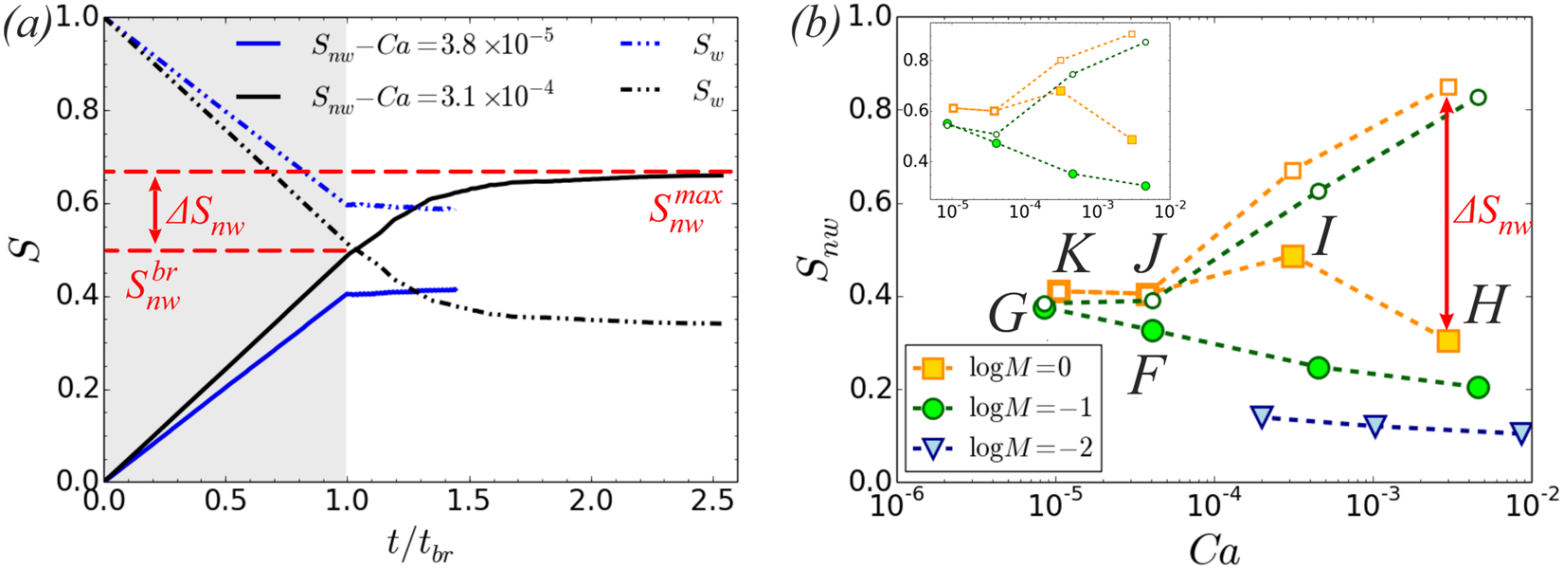
(**a**) Saturation (*S*_nw_: solid lines, *S*_*w*_: dash-dotted lines) versus time in scaled units for *logM* = 0 and *Ca* = 3.1 × 10^−4^, *Ca* = 3.8 × 10^−5^. The shaded area corresponds to the regime of linear increase (decrease) of *S*_nw_(*S*_w_) with time (*t* ≤ *t*_*br*_) as non-wetting phase is injected at a constant injection flow rate up to ${S}_{{\rm{nw}}}^{br}$. Saturation then converges to maximum achievable non-wetting phase saturation, ${S}_{{\rm{nw}}}^{max}$, if injection continues. (**b**) The non-wetting phase saturation at breakthrough, ${S}_{{\rm{nw}}}^{br}$, (filled symbols) and maximum achievable non-wetting phase saturation, ${S}_{{\rm{nw}}}^{max}$, (empty symbols) as a function of *Ca* and varying *M*. Inset: Results for the subdomain *x* ≤ 1.9 *mm*.

If injection continues, beyond the time the non-wetting phase reached the outlet (*t* = *t*_*br*_), then *S*_nw_ may increase further (up to maximum achievable ${S}_{{\rm{nw}}}^{max}$) depending on the inlet/outlet pressure difference Δ*P*. At high *Ca* the injected phase will continue to displace the defending fluid due to the high Δ*P* (case *Ca* = 3.1 × 10^−4^ in Fig. 5(a)), while for low *Ca* no further increase in *S*_nw_ will be observed (case *Ca* = 3.8 × 10^−5^ in Fig. 5(a)), as the fluid will flow through the formed displacement pathways. Hence, once capillary fingering dictates the displacement process no significant change in *S*_nw_ is achieved beyond ${S}_{{\rm{nw}}}^{br}$ (${S}_{{\rm{nw}}}^{br}\simeq {S}_{{\rm{nw}}}^{max}$). This is evident from results shown in Fig. 5. Moreover, capillary fingering displacement commences at a higher *Ca* for $\mathrm{log}\,M=0$ (*Ca* ~ 4 × 10^−5^) than for $\mathrm{log}\,M < 0$ (*Ca* ~ 9 × 10^−6^ for $\mathrm{log}\,M=-\,1$), as expected. Here we assume capillary fingering displacement to be commencing as ${\rm{\Delta }}{S}_{{\rm{nw}}}={S}_{{\rm{nw}}}^{max}-{S}_{{\rm{nw}}}^{br}\to 0$. Irrespective of the viscosity ratio though, the limiting value for ${S}_{{\rm{nw}}}^{max}$ seems to remain the same (${S}_{{\rm{nw}}}^{max}\sim 0.4$) for results in the capillary fingering regime. In order to minimize the impact of capillary end effects on ${S}_{{\rm{nw}}}^{max}$, we also examine *S*_nw_ in the first subvolume of the rock, indicated with the red arrow in Fig. 4(a) (*x* ≤ 1.9 *mm*). This is shown in the inset of Fig. 5(b) and demonstrates the same qualitative behavior.

**Figure 6:**
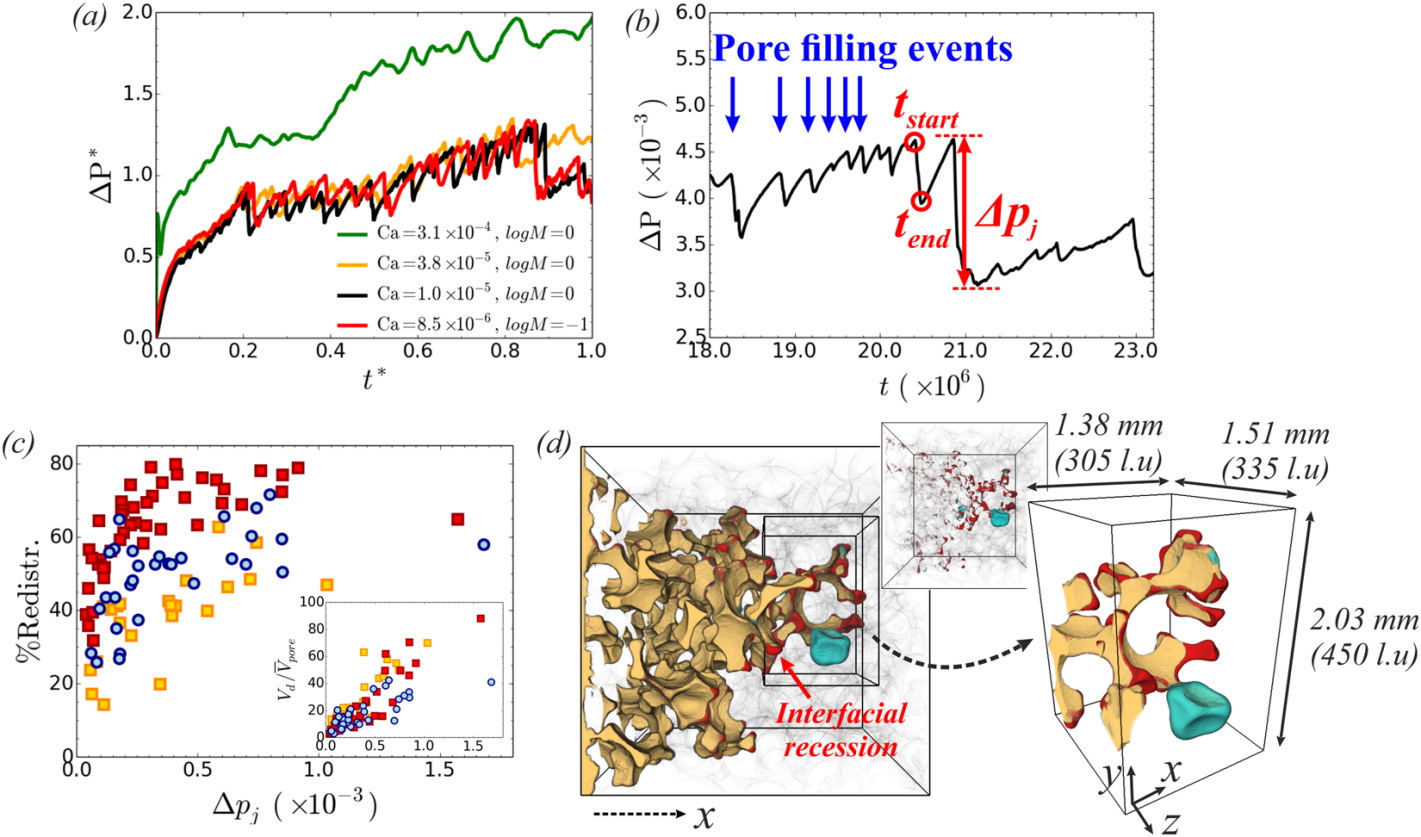
(**a**) Inlet-outlet pressure difference as a function of time in scaled units. Pressure is normalized by the average capillary entry pressure (using the average radius of invaded throats) and time by the breakthrough time, *t*_*br*_. A decrease is observed in ΔP* as *Ca* decreases from *Ca* = 3.1 × 10^−4^ (Fig. 2I) to 3.8 × 10^−5^ (Fig. 2J). Decreasing the injection flow rate further (and consequently *Ca*) does not seem to have a big impact on ΔP* and results within the capillary fingering regime show overlapping data for ΔP*. (**b**) We identify the pore filling events from the pressure signal. (**c**) Degree of fluid redistribution for results in the capillary fingering regime: (i) *logM* = 0 (squares) *Ca* = 3.8 × 10^−5^ (orange, Fig. 2J), *Ca* = 1.0 × 10^−5^ (red, Fig. 2K), and (ii) *logM* = −1, *Ca* = 8.5 × 10^−6^ (blue circles, Fig. 2G). Inset: The draining volume, normalized by the average pore volume, as a function of pressure drop Δ*p*_*j*_. (**d**) Haines jump event and the associated fluid rearrangement from a simulation at *logM* = −1, *Ca* = 8.5 × 10^−6^. The region occupied by the non-wetting phase that remains unchanged before and after the jump event is shown in yellow, while the draining pore body (*V*_*d*_) and interfacial recession (*V*_*imb*_) is shown in light blue and red respectively. The interfacial energy created corresponds to 56% of the total available energy (externally performed work of pressure and the energy released at the imbibition sites), while the volume of fluids being redistributed corresponds to 71% of the draining volume.

Our results agree with the ones reported in the literature^3,6,7,12^, i.e. the maximum achievable displacement efficiency decreases with decreasing *Ca* and as we move from stable displacement to capillary fingering and viscous fingering. Another important observation is that the saturation at breakthrough ${S}_{{\rm{nw}}}^{br}$ (filled symbols) generally increases with decreasing *Ca* (see Fig. 5(b)), in agreement with numerical results by Tsuji *et al*.^10^, who investigated drainage in Berea sandstone using a color gradient LB approach^41^. Interestingly though, this is not the case for $\mathrm{log}\,M=0$ as *Ca* decreases from *Ca* = 3.1 × 10^−4^ (Fig. 2I) to 3.8 × 10^−5^ (Fig. 2J). On the contrary a significant decrease in ${S}_{{\rm{nw}}}^{br}$ is observed (~−17% in the whole domain and ~−12% in the first subvolume region, *x* ≤ 1.9 *mm*). Intuitively it would be reasonable to expect that ${S}_{{\rm{nw}}}^{br}$ should increase due to capillary fingering controlling the displacement process. What comes into play, going from *Ca* = 3.1 × 10^−4^ (Fig. 2I) to 3.8 × 10^−5^ (Fig. 2J), is the onset of Haines jumps, which becomes profound from the flow field and pressure drop shown in Fig. 3(b,c). On the other hand a decrease is observed in the inlet/outlet pressure difference Δ*P*, as shown in Fig. 6(a), that could justify the decrease in ${S}_{{\rm{nw}}}^{br}$. Hence, the question to be addressed is whether this reduction is due to: i) the onset of Haines jumps or ii) the lower pressure difference driving the fluid flow for the lowest *Ca* case (Fig. 6(a)), which makes it difficult for the non-wetting phase to access regions of the pore matrix due to their higher capillary entry pressure. This will be addressed in the next section.

### Haines jumps, fluid rearrangement and displacement pathways

To examine the impact of Haines jumps on the displacement efficiency, we analyze the events from simulations in the capillary fingering regime: i) *logM* = 0: *Ca* = 3.8 × 10^−5^ (Fig. 2J), *Ca* = 1.0 × 10^−5^ (Fig. 2K) and ii) *logM* = −1: *Ca* = 8.5 × 10^−6^ (Fig. 2G). Particular focus is given on the fluid redistribution associated with the events and how this can affect the displacement process. As demonstrated experimentally^19,21^ and numerically^27,42^, Haines jumps are associated with both drainage and imbibition dynamics, as wetting phase imbibes surrounding regions of the jump, displacing the non-wetting phase and providing so a significant volume of non-wetting phase to the draining pores. To assess this quantitatively, we identify the pore filling events from the pressure signal, see Fig. 6(b). Using the fluids’ configuration at the beginning (*t*_*start*_) and the end (*t*_*end*_) of each event we quantify the degree of fluid redistribution *R*_%_, as we can measure directly the draining volume *V*_*d*_, as well as the non-wetting phase volume originating from the imbibition sites *V*_*imb*_. The degree of fluid redistribution, defined as *R*_%_ = *V*_*imb*_/*V*_*d*_, is shown in Fig. 6(c). As the injection flow rate (*Ca*) decreases for *logM* = 0 (*J* to *K*), *R*_%_ increases from *R*_%_ ~ 39 ± 12% up to even *R*_%_ ~ 80% (63 ± 12%). Decreasing the viscosity ratio to *logM* = −1 (*K* to *G* by increasing *η*_*w*_ and keeping ${{\rm{u}}}_{{\rm{nw}}}^{inj}$ fixed) results in a decrease in *R*_%_ (50% ± 12%), reflecting the increased viscous dissipation rate in the wetting phase in *G*. Nevertheless results verify that extensive fluid redistribution takes place, in line with the experimental findings that the required draining volume can not be supported by the externally imposed flow rate in the time scale of the jump^19,21^. Berg *et al*.^19^ report *R*_%_ ~ 99% in Berea sandstone at even lower *Ca* ~ 10^−8^. Moreover, as expected, a correlation exists between the magnitude of the pressure drop Δ*p*_*j*_ and the draining volumes *V*_*d*_, see inset of Fig. 6(c). The fluid redistribution associated with a jump event at *logM* = −1, *Ca* = 8.5 × 10^−6^ (Fig. 2G) can be visualized in Fig. 6(d). The region occupied by the non-wetting phase that remains unchanged during the event is shown in yellow, while (i) the draining pore and (ii) locations of interfacial recession (imbibition sites) are shown in light blue and red respectively. Similar interfacial recession patterns were observed in experiments by Andrew *et al*.^43^ during drainage of Ketton limestone.

A rough estimate for the time scale of the interfacial jumps, Δ*t*_*j*_ = *t*_*end*_ − *t*_*start*_, considering the capillary forces due to differences in the interfacial curvature and the mass of the accelerated fluid^25^, gives ${\rm{\Delta }}{t}_{j}\sim \beta {(\rho {r}_{pore}^{3}/\gamma )}^{\mathrm{1/2}}\simeq \beta \times {10}^{3}$ l.u, where *β* > 1 is a dimensionless constant that depends on the pore shape. This analysis neglects the viscous resistance to the flow; hence it is justifiable to obtain a higher time scale in the simulations. Results are an order of magnitude higher for *logM* = 0 (Δ*t*_*j*_ = 4.5 ± 2.4 × 10^4^ − *Ca* = 3.8 × 10^−5^, Δ*t*_*j*_ = 6.5 ± 4.9 × 10^4^ − *Ca* = 1.0 × 10^−5^), while as the viscous resistance in the wetting phase increases further for *logM* = −1, *Ca* = 8.5 × 10^−6^, we obtain Δ*t*_*j*_ = 10.5 ± 5.2 × 10^4^.

**Figure 7:**
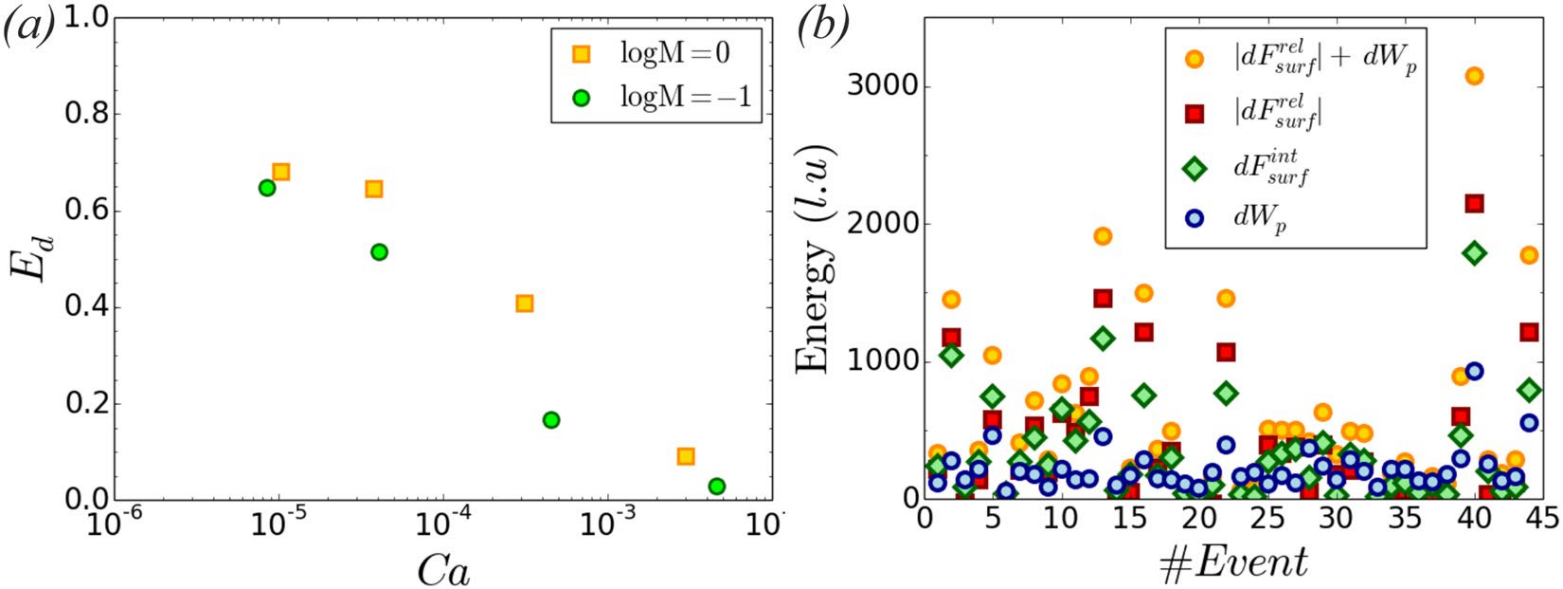
(**a**) Efficiency of the conversion of the work of drainage to surface energy for varying *Ca* and viscosity ratio. We estimate the change in surface energy Δ*F*_*surf*_ and the pressure-volume work *dW*_*p*_ done on the system at *t* = *t*_*br*_. (**b**) Analysis of Haines jump events from a simulation at $\mathrm{log}\,M=0$, *Ca* = 1.0 × 10^−5^ (Fig. 2K) and the corresponding surface energies. We measure the surface areas using a robust tool in Matlab^60^. The available energy at each event which accelerates the fluids and generates new interfaces originates at a big extend from wetting at the imbibition sites, $d{F}_{surf}^{rel}=\gamma \,\cos \,{\theta }^{eq}d{A}_{ns}$, besides the externally provided pressure-volume work *dW*_*p*_.

From an energy point of view, during the drainage process surface energy is stored in the system due to the externally performed work of pressure, ${W}_{p}=\int \,{\rm{\Delta }}PQdt$. However, due to viscous dissipation, the change in surface energy Δ*F*_*surf*_ is less than the work done on the system^44^. This is shown in Fig. 7(a) where we plot the efficiency of the conversion of work of drainage (pressure-volume work) to surface energy, *E*_*d*_ = Δ*F*_*surf*_/*W*_*p*_, as a function of *Ca*. At the highest *Ca* a significant amount of energy is also converted to kinetic energy *E*_*k*_ ≥ 0. As the injection flow rate drops 3 orders in magnitude (${{\rm{u}}}_{{\rm{nw}}}^{inj}:{10}^{-3}\to {10}^{-6}$), *E*_*k*_ becomes negligible and the energy input into the system is converted mainly to surface energy and dissipated. *E*_*d*_ increases as *Ca* (injection flow rate) decreases due to lower viscous dissipation rate. Seth and Morrow^44^ estimate *E*_*d*_ as a function of wetting phase saturation *S*_*w*_ in centriguge experiments, but do not quantify the importance of viscous forces to surface tension (*Ca*). They report *E*_*d*_ ~ 55% for Berea sandstone at *S*_*w*_ ~ 0.6, which increases to *E*_*d*_ ~ 90% for sphere packs. Our results at this value of *S*_*w*_ reveal *E*_*d*_ ~ 68% for the lowest *Ca* simulations. In the capillary fingering regime, energy stored in the menisci and the fluid columns of the non-wetting phase in the pore throats is released during the jump events, converted into kinetic energy and interfacial energy and dissipated. Hence, the system is essentially using existing energy already stored as surface energy to support these abrupt events and the fluid redistribution, while the fluids acceleration increases the energy losses due to the higher viscous dissipation. The change in surface energy is given by *dF*_*surf*_ = *γdA*_*int*_ + *γ*_*ws*_*dA*_*ws*_ + *γ*_*ns*_*dA*_*ns*_, where *dA*_*int*_, *dA*_*ws*_ and *dA*_*ns*_ are the increments of the areas of the fluid-fluid, solid-wetting phase fluid and solid - non wetting phase fluid interfaces respectively and *γ*, *γ*_*ws*_, *γ*_*ns*_ the corresponding surface tensions. Given that *dA*_*ns*_ = −*dA*_*ws*_, this can be expressed as *dF*_*surf*_ = *γ*(*dA*_*int*_ + cos *θ*^*eq*^*dA*_*ns*_). Figure 7(b) presents results from *logM* = 0, *Ca* = 1.0 × 10^−5^ (Fig. 2K) with the energy released due to wetting, $d{F}_{surf}^{rel}=\gamma \,\cos \,{\theta }^{eq}d{A}_{ns} < 0$, the work done on the system during the time scale of the jumps, $d{W}_{p}=\int {\rm{\Delta }}PQdt$, as well as the corresponding energy of the newly created interfaces, $d{F}_{surf}^{int}=\gamma d{A}_{int}$. This reveals that $d{F}_{surf}^{rel}$ provides a significant fraction of the energy needed to support the Haines jumps (57 ± 21% of the overall available energy $|d{F}_{surf}^{rel}|+d{W}_{p}$). The interfacial areas generated correspond to 59 ± 17% of the overall available energy, with the rest of the energy mainly dissipated. For case *logM* = −1, *Ca* = 8.5 × 10^−6^ (Fig. 2G) the interfacial areas generated correspond to 57% ± 12% of the overall available energy.

**Figure 8:**
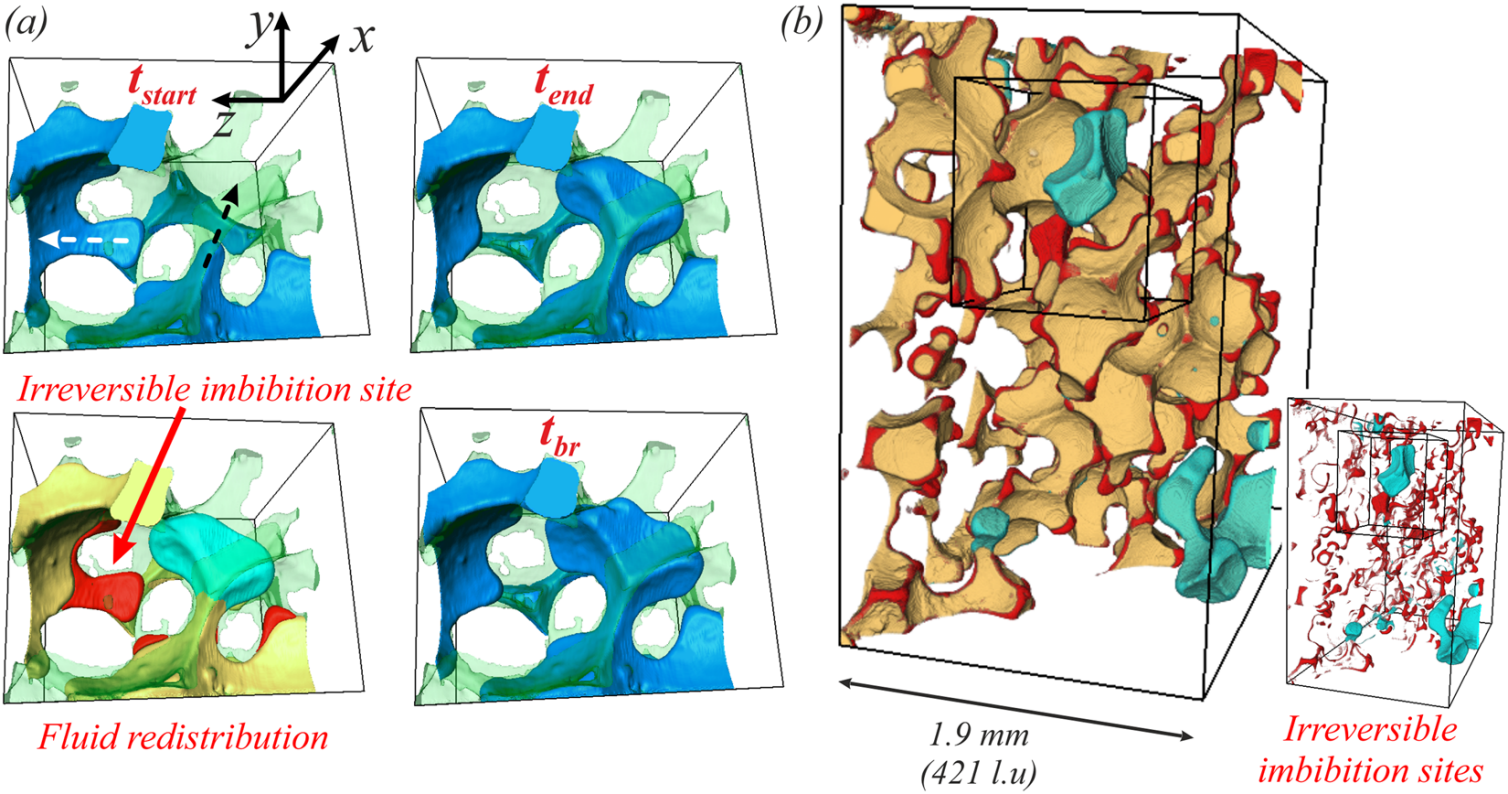
Irreversible fluid redistribution - Results at *Ca* = 8.5 × 10^−6^ and $\mathrm{log}\,M=-\,1$ (Fig. 2G): (**a**) The non-wetting phase configuration (blue) at the beginning and the end of a jump event, as identified from the pressure signal (*t*_*start*_ = 18.18 × 10^6^ − ${t}_{start}^{\ast }=0.854$, *t*_*end*_ = 18.35 × 10^6^ − ${t}_{end}^{\ast }=0.862$), as well as the corresponding fluid redistribution. The region occupied by the non-wetting phase prior the jump but not after is shown in red (imbibition sites). Subsequent events don’t change the occupancy of this pore space and the wetting phase becomes eventually trapped there (irreversible imbibition displacement), see configuration at breakthrough (*t* = *t*_*br*_). (**b**) Total irreversible imbibition sites, comparing the fluids’ distribution at *t* = 18.18 × 10^6^ (prior a jump, t* = 0.856) and *t* = 21.15 × 10^6^ (before breakthrough, t* = 0.996). The region occupied by the non-wetting phase that remains unchanged is shown in yellow, while the draining regions and interfacial recession sites (irreversible displacement) are shown in light blue and red respectively.

Examining the impact of Haines jumps on the displacement efficiency revealed that these events can potentially decrease the displacement efficiency if the fluid redistribution at the imbibition sites can be characterized as irreversible. This irreversibility means that after the jump the non-wetting phase never manages to displace the wetting phase back at the imbibition sites, see Fig. 8(a) with results from a simulation at *logM* = −1, *Ca* = 8.5 × 10^−6^ (Fig. 2G). The wetting phase eventually becomes trapped there (*t* = *t*_*br*_). It was observed in our previous work in micromodels^27^ (see Fig. 11 therein) that reversal of the flow (imbibition) can happen even after the non-wetting phase passed the narrowest restriction point in the throat (hence exceeded the capillary entry pressure) or even escaped in the wider pore body. Therefore, it is not only the volume of the throats occupied by the non-wetting phase prior the event that matters, but most importantly the fact that the displacement pathways can be affected; this may have a bigger impact on the displacement efficiency as regions of the pore matrix become inaccessible to the non-wetting phase. Comparing the fluids’ distribution prior an event and just before breakthrough, enables us to identify the total locations that can be characterized by irreversible displacement. This is illustrated in Fig. 8(b) (regions in red). The volume of these regions corresponds to a loss of ~4% of the existing pore space occupied by the non-wetting phase at the beginning of the jump event.

**Figure 9:**
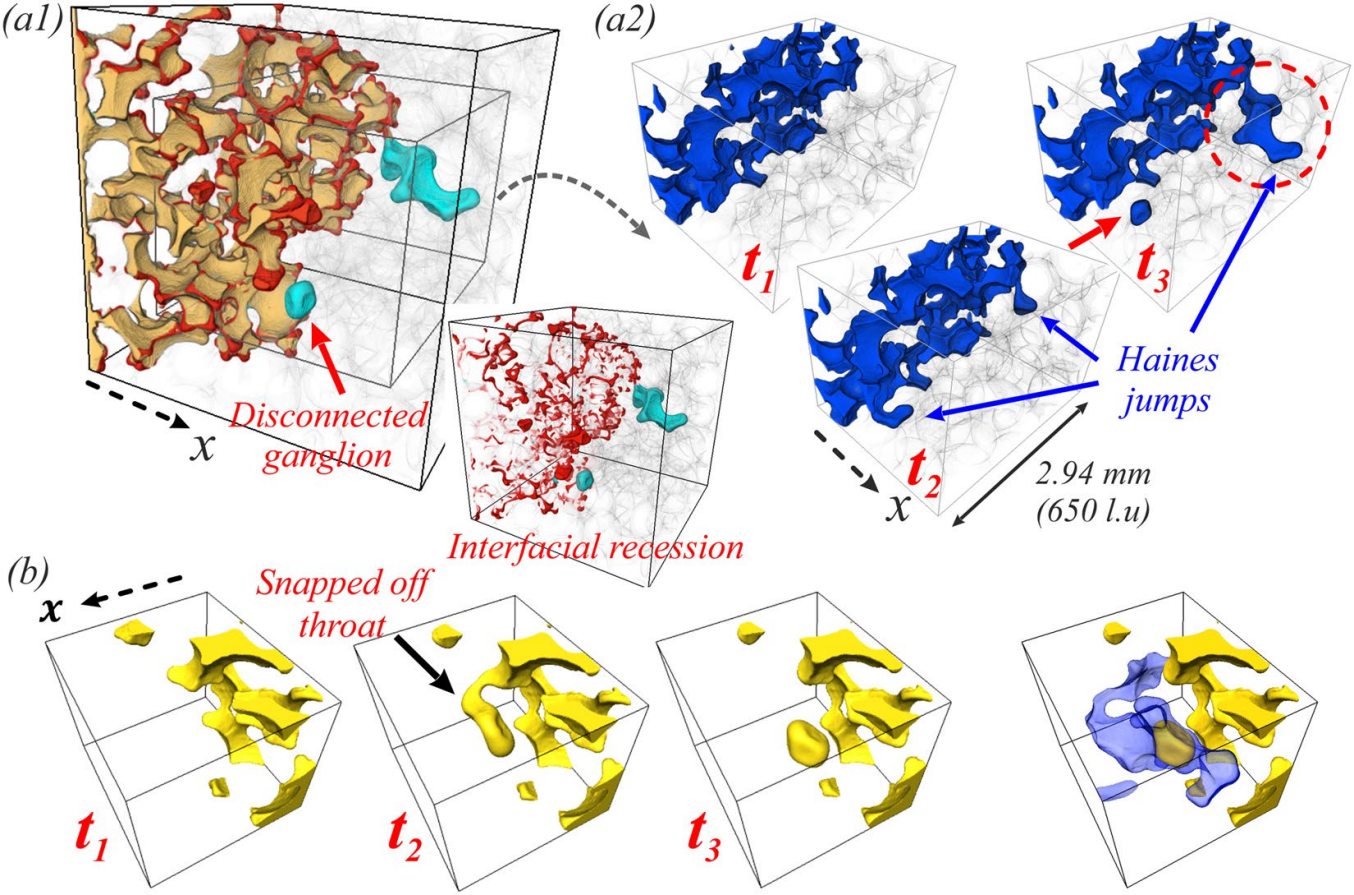
Distal snap-off. Results from a simulation at *Ca* = 1.0 × 10^−5^ and $\mathrm{log}\,M=0$ (Fig. 2K). (a1) Fluid rearrangement associated with the jump events (*V*_*imb*_/*V*_*d*_ = 64.8%). (a2) Multiple jump events lead to distal snap-off (red arrow)^43^ (${t}_{1}^{\ast }=0.892$, ${t}_{2}^{\ast }=0.894$, ${t}_{3}^{\ast }=0.900$). The jump event indicated in red cycle proceeds in a cascadelike manner over multiple geometrically defined pores resulting in a big reduction in capillary pressure even in throats that are a significant distance away from the jump event. The reduction in the capillary pressure in this throat below its snap-off capillary pressure causes disconnection of the non-wetting phase (distal snap-off). (**b**) Same distal snap-off event from a different viewing angle. The region in light blue on the right panel is pore space that, at breakthrough, is occupied by the non-wetting phase for *Ca* = 3.1 × 10^−4^ and $\mathrm{log}\,M=0$ (Fig. 2I).

Another mechanism and important evidence of how Haines jumps can potentially lead to a decrease in the displacement efficiency comes from analysing an event at *logM* = 0, *Ca* = 1.0 × 10^−5^ (Fig. 2K), see Fig. 9. Initially two jump events take place in the two locations shown in Fig. 9(a2) at *t* = *t*_2_. The jump event, indicated in red cycle, proceeds in a cascade like manner over multiple geometrically defined pores, causing a big reduction in capillary pressure even in throats that are a significant distance away from the jump event. The reduction in the capillary pressure in this throat below its snap-off capillary pressure causes the disconnection of the non-wetting phase and produces a long-lasting fluid configuration as it remains disconnected until the end of the simulation. Distal snap-off events may have a bigger impact on the flow than local snap-off events, and have been observed experimentally for CO_2_-brine system^43^. Here for example, due to the snap-off event, the injected phase loses access to the pore space shown in light blue in Fig. 9(b), which is filled by the non-wetting phase for $\mathrm{log}\,M=0$, *Ca* = 3.1 × 10^−4^ (Fig. 2I). Therefore, such events can firstly affect the displacement pathways and potentially lead to a decrease in the displacement efficiency.

The above two mechanisms demonstrate how Haines jumps potentially affect the displacement efficiency. Irrespective of the type of Haines jumps, whether backward or forward, these abrupt events decrease the storage efficiency of the non-wetting phase, contrary to what is argued by Yamabe *et al*.^12^. Their findings refer to the impact of the jump events on CO_2_ saturation per injection depth, similar to what is shown in Fig. 4, but do not actually examine the impact of Haines jumps on storage efficiency, nor the aspect of fluid rearrangement. Furthermore, an important remark to make here is that using a constant injection flow rate (velocity boundary conditions) to drive the fluid flow enables the study of the low *Ca* flow regime characterised by Haines jumps. Even as the injection flow rate decreases, pressure slowly builds up in the system until the capillary entry pressure is overcome. This would not have been possible if a body force (pressure gradient) is used, as the injected phase may not be able to reach the outlet of the domain if the overall pressure difference generated does not overcome the required capillary entry pressure along the percolation pathway^9,10,29^.

## Conclusions

Drainage displacement patterns have been identified in the pioneering work of Lenormand *et al*.^3^, as capillary fingering (low *Ca*), viscous fingering (high *Ca*, $\mathrm{log}\,M < 0$) and stable displacement (high *Ca*, $\mathrm{log}\,M > 0$). These distinctively different displacement flow regimes can affect significantly the displacement efficiency, which is defined as the fraction of the defending wetting fluid that has been displaced from the pore matrix when the injected non-wetting phase reached the outlet of the domain. A more important feature is the maximum achievable displacement efficiency, established by continuing the injection until saturation convergence is achieved.

Here, in order to understand these pore-scale displacement phenomena and their impact on CO_2_ storage efficiency, we investigate the immiscible displacement (drainage) of a wetting fluid in a porous medium by a non-wetting fluid using multi-GPU free energy lattice Boltzmann simulations, under various capillary numbers *Ca* and viscosity ratios *M*. As a first step we first verify the existence of the three typical fluid displacement patterns, before turning our attention on the impact of these processes on storage efficiency. Our results agree with the ones reported in the literature^3,6,12^, i.e. maximum achievable displacement efficiency decreases with decreasing *Ca* and as we move from stable displacement to capillary fingering and viscous fingering.

Then we focus on flow at low *Ca* (capillary fingering regime), and the impact of Haines jumps on displacement efficiency, by decreasing the injection flow rate. During these sharp interfacial jumps pressure drops abruptly. Pressure drop coincides with the sharp increase of the non-wetting phase velocity. Extensive fluid rearrangement, which increases as *Ca* decreases, provides extra non-wetting phase needed for draining wider pore bodies. We identify two possible mechanisms that potentially affect the displacement process: i) irreversible imbibition displacement and ii) distal snap-off events. Both can render regions of the pore space inaccessible to the injected non-wetting phase. During the redistribution of the fluids, wetting phase flowing in the direction opposite to the mean direction of the flow displaces the non-wetting phase. If this imbibition displacement is irreversible, it can lead to wetting phase trapped due to subsequent jump events, but most importantly leads to an overall decrease in the displacement efficiency by subsequently blocking access to other regions of the pore space. Distal snap-off events can also change the displacement process, as the disconnection of the non-wetting phase may prohibit the injected phase from accessing regions of the porous matrix. From an energy point of view, the extensive fluid redistribution associated with the events and the increased viscous dissipation rate as the fluids accelerate, mean that less energy is eventually stored in the system as surface energy; hence this decreases the efficiency of the conversion of work of drainage to surface energy.

Hence, our findings have important implications in the context of geological sequestration of CO_2_, as Haines jumps become a limiting factor in the storage process. Suppressing these events, for example by decreasing surface tension through the use of surfactants, can shift the flow regime to higher capillary numbers and increase considerably the saturation of CO_2_ that can be attained in the porous matrix. Eventually, it would be desirable to restore surface tension to its original value, e.g. adsorption of surfactants at solid surfaces or injection of brine to dilute the concentration of surfactants, as high capillary forces are needed to trap CO_2_ in the pore space over long time scales. The latter can be in line with water-alternating-gas injection schemes^45^ and the findings of Herring *et al*.^38^ who demonstrated that both the drainage (CO_2_ injection) and imbibition (subsequent water injection) processes can be engineered in order to maximize residual trapping within the permeable medium.

## Methods

### Porous rock

The three dimensional geometry used for the simulations was reconstructed from micro-CT images of Ketton limestone (porosity 0.159 and permeability 9.4 Darcy)^30^. We note that the Ketton limestone has microporous grains with micro-pore sizes well below the resolution of the CT scan^46^ and this is neglected in the flow simulations presented in the paper. However, given that we expect the permeabilities of the microporosity and macroporosity regions to be several orders of magnitude apart, and that we are considering capillary pressures below the entry pressure of the micro-porous regions, the proposed approach to simulate flow in the pore space that corresponds to the macroporosity only is reasonable.

### Numerical Model - Free energy lattice Boltzmann method

Here we describe the numerical method we use, covering the thermodynamics, the dynamical equations of motion and finally the numerical implementation. We have chosen to use a standard free energy lattice Boltzmann approach^32^ to solve the hydrodynamic equations of motion for two-phase flow directly on micro-CT images of porous media. The method belongs to a class of hydrodynamic models, called diffuse interface (phase field) models^47–51^, where the sharp-interface formulation is replaced with a continuous variation of an order parameter over a finite-sized interfacial region. These models introduce a diffusive mechanism at the interfacial region, which regularizes the viscous dissipation singularity^52^, and allows the contact line to slip on a solid substrate.

#### Thermodynamics of the fluid

The equilibrium properties of a binary fluid can be described by a Landau free energy functional^32^

1 $$ {\mathcal F} ={\int }_{V}\,({f}_{b}+\frac{{\kappa }_{\varphi }}{2}{({\partial }_{\alpha }\varphi )}^{2})\,dV+{\int }_{S}\,{f}_{s}\,dS.$$

The first term in the integrand is the bulk free energy density given by

2 $${f}_{b}=-\,\frac{A}{2}{\varphi }^{2}+\frac{A}{4}{\varphi }^{4}+\frac{{c}^{2}}{3}\rho \,\mathrm{ln}\,\rho ,$$

where *ϕ* is the concentration or order parameter, *ρ* is the fluid mass density and *c* is a lattice velocity parameter. *A* is a constant with dimensions of energy per unit volume. The bulk free energy density *f*_*b*_ has minima at *ϕ*_*eq*_ = ±1, corresponding to the two bulk fluid phases, while the locus *ϕ* = 0 is chosen as the position of the interface. The term in *ρ* controls the compressibility of the fluid^53^.

The gradient term, $\frac{{\kappa }_{\varphi }}{2}{({\partial }_{\alpha }\varphi )}^{2}$, accounts for the excess free energy due to the presence of interfaces by penalizing spatial variations of the order parameter *ϕ*. This gives rise to the interface tension $\gamma =\sqrt{8{\kappa }_{\varphi }A/9}$ and to the interface width $\xi =\sqrt{{\kappa }_{\varphi }/A}$^32^.

The affinity of the fluids to solid surfaces is controlled by the final term in the free energy functional, eq. 1. Following Cahn^54^, the surface energy density is taken to be of the form *f*_*s*_ = −*hϕ*_*s*_, where *ϕ*_*s*_ is the value of the order parameter at the surface. Minimisation of the free energy gives an equilibrium wetting boundary condition^32^

3 $${\kappa }_{\varphi }\,{\partial }_{\perp }\varphi =-\,\frac{d{f}_{s}}{d{\varphi }_{s}}=-\,h.$$

The value of the parameter *h* (the surface excess chemical potential) is related to the equilibrium contact angle *θ*^*eq*^ via^32^

4 $$h=\sqrt{2{\kappa }_{\varphi }A}\,{\rm{sign}}\,[\frac{\pi }{2}-{\theta }^{{\rm{eq}}}]\,\sqrt{\cos \,(\frac{\alpha }{3})\,\{1-\,\cos \,(\frac{\alpha }{3})\}},$$

where $\alpha =\arccos \,{(\sin }^{2}\,{\theta }^{eq})$ and the function sign returns the sign of its argument.

This choice of the free energy leads to the chemical potential

5 $$\mu =\frac{\delta  {\mathcal F} }{\delta \varphi }=-\,A\varphi +A{\varphi }^{3}-{\kappa }_{\varphi }\,{\partial }_{\gamma \gamma }\varphi ,$$

and the pressure tensor^47^

6 $${P}_{\alpha \beta }=[{p}_{b}-{\kappa }_{\varphi }\,\varphi {\partial }_{\gamma \gamma }\varphi -\frac{{\kappa }_{\varphi }}{2}{({\partial }_{\gamma }\varphi )}^{2}]{\delta }_{\alpha \beta }+{\kappa }_{\varphi }({\partial }_{\alpha }\varphi )({\partial }_{\beta }\varphi ),$$

where ${p}_{b}=\frac{{c}^{2}}{3}\rho -\frac{1}{2}A{\varphi }^{2}+\frac{3}{4}A{\varphi }^{4}$ is the bulk pressure.

#### Equations of motion

The hydrodynamic equations for the system are the continuity, eq. 7, and the Navier-Stokes, eq. 8, equations for a nonideal fluid

7 $${\partial }_{t}\rho +{\partial }_{\alpha }(\rho {u}_{\alpha })=0,$$

8 $${\partial }_{t}(\rho {u}_{\alpha })+{\partial }_{\beta }(\rho {u}_{\alpha }{u}_{\beta })=-\,{\partial }_{\beta }{P}_{\alpha \beta }+{\partial }_{\beta }[\eta ({\partial }_{\beta }{u}_{\alpha }+{\partial }_{\alpha }{u}_{\beta })],$$

where **u**, **P**, *η* are the fluid velocity, pressure tensor and dynamic viscosity respectively. The above equations are coupled with a convection-diffusion equation,

9 $${{\rm{\partial }}}_{t}\varphi +{{\rm{\partial }}}_{\alpha }(\varphi {u}_{\alpha })={M}_{\varphi }{{\rm{\nabla }}}^{2}\mu ,$$

that describes the dynamics of the order parameter *ϕ*. *M*_*ϕ*_ is a mobility coefficient.

#### Lattice Boltzmann method

The equations of motion are simulated using a standard free energy lattice Boltzmann algorithm for a binary fluid^32^. In particular we use a three dimensional model with 19 discrete velocity vectors (D3Q19) and adopt a multiple relaxation time (MRT)^55^ approach for the evolution of the distribution functions ${f}_{i}^{^{\prime} }s$ associated with eqns 7 and 8. Details of the implementation of the Lattice Boltzmann algorithm are given in the references^32,33,56,57^ and are not repeated here. We comment that, within the lattice Boltzmann framework, the time-step is set to *dt* = 1. There is no need to change the time-stepping depending on the value of *Ca* as the relevant time scales for the instability under investigation are much larger than the lattice Boltzmann time-step (time scale of the pressure drop ~10^4^ l.u).
